# Asset Administration Shell Tool Comparison: A Case Study with Real Digital Twins Used in Petrochemical Industry

**DOI:** 10.3390/s25071978

**Published:** 2025-03-22

**Authors:** Fatih Kaya, Ezgi Şanlı, Özlem Albayrak, Perin Ünal, Pinar Kirci

**Affiliations:** 1 Department of Computer Engineering, Uludağ University, 16059 Bursa, Turkey; pinarkirci@uludag.edu.tr; 2 TEKNOPAR, 06378 Ankara, Turkey; sanli@teknopar.com.tr (E.Ş.); or albayrak@teknopar.com.tr (Ö.A.); punal@teknopar.com.tr (P.Ü.); 3 Department of Software Engineering, TED University, 06420 Ankara, Turkey

**Keywords:** digital twin, Asset Administration Shell, Industry 4.0, digital twin interoperability

## Abstract

Being a cornerstone of Industry 4.0, Asset Administration Shell (AAS) enables seamless integration and interaction among the physical and digital worlds. There are multiple different tools and technologies available for implementing AAS. The purpose of this study is to support the tool and technology selection decision of AAS modelers and implementers. For that purpose, we conducted a literature survey and identified four active tools, and in the study, we included all of them: AASX server, Eclipse BaSyx, FA^3^ST service, and NOVAAS. Using a comprehensive criteria list, we conducted a thorough comparison of the selected technologies. The comparison was made in two steps: first for initial learning exercises and second for a real case study where digital twins belong to real assets in a facility belonging to the petrochemical industry. Among the evaluated tools, Eclipse BaSyx demonstrated superior performance compared to the other three tools investigated in this study. Future research will focus on incorporating machine learning (ML) and deep learning (DL) models associated with the assets, leveraging datasets generated by the sensors installed on the system.

## 1. Introduction

The accelerated dissemination of digital twin utilization requires seamless integration of smart, interconnected components into the production environment, forming the basis of Industry 4.0. Such integrations necessitate clear guidelines and a standardized framework, which leads to the globally recognized principles of Industry 4.0. In line with these standards, the same data can be deployed in multiple domains for different purposes and can be transformed for interoperability, communication, and information exchange [1]. To address standardization requirement, the Reference Architecture Model Industrie 4.0 (RAMI 4.0) has been presented [2]. An identical framework for Industrie 4.0 is provided by RAMI 4.0, ensuring that all parties involved share the same language. RAMI 4.0 facilitates the smooth integration, real-time monitoring, and optimization of industrial processes by establishing a connection between digital representations and physical assets via Asset Administration Shell (AAS).

AAS is able to incorporate passive assets like tools or components in addition to active assets like machines. According to their capabilities, there are three types of AASs in three different types: Type 1 is called passive AAS, which makes it easier to understand the content of each file in a predefined structure and configures asset data for straightforward retrieval and inspection. It does not support real-time communication and is best suited for scenarios where assets need to share structured information without direct interaction. Type 2 and Type 3 provide more interactive functionalities that enable dynamic data exchange with AAS files, with Type 3 additionally supporting broader Industry 4.0 communication methods, whereas Type 2 relies exclusively on APIs [3,4]. Type 2, called reactive AAS, is the version used in this article in the examples, and it provides access to its data through an API and enables external systems to query and retrieve information dynamically, making it useful in environments where assets need to respond to incoming requests but do not initiate communication themselves. Proactive AAS, as Type 3, goes a step further by not only responding to queries like Type 2 but also actively initiating interactions with other Industry 4.0 components. This allows for real-time collaboration, automated decision making, and synchronized operations in complex industrial environments such as smart manufacturing or autonomous production systems.

In some circumstances, it is required to save a file on the server to make the data permanent or modify sub-elements of the AAS with associated values, which means that a file format needs to be defined that can be used to store and access these data on the server. Therefore, the generic package file format includes the Asset Administration Shell structure, which is defined as AASX [4].

The need for standardized data storage and exchange formats like AASX becomes even more critical as Industry 4.0 continues to evolve. Various approaches have been introduced to address challenges such as semantic interoperability and digital twin frameworks, each playing a role in automation, lifecycle integration, and other key application areas. With the increasing adoption of these technologies, the digital twin concept is gaining momentum, providing a virtual representation of physical assets to enhance monitoring, optimization, and decision-making processes.

The articles [5,6,7] broadly focused on creating adaptable Industry 4.0 contexts to achieve flexible and data-driven industrial systems by showcasing the role of AAS-based models in unifying heterogeneous components. The use of AAS to improve maintenance and lifecycle processes by providing a unified, standardized representation of assets enhancing overall system performance and reliability was a key focus of these studies [8,9,10,11,12].

In parallel with these advancements, efforts to improve interoperability and data exchange in AAS adoption remain another issue. The studies [13,14] focused on automated data conversion, introducing an API-based file conversion service and a framework for semantic interpretation to transform AAS data. Meanwhile, other studies [15,16,17] have addressed interoperability by developing mapping solutions between AAS, OPC UA, and proprietary formats using structured transformation rules.

On the other hand, open-source solutions play a crucial role in the widespread adoption and evolution of Industry 4.0 technologies, including AAS. By providing accessible, transparent, and adaptable solutions, open-source initiatives foster collaboration among industry leaders, researchers, and developers, accelerating innovation and standardization. They enable cost-effective implementation, reducing dependency on proprietary systems and enhancing interoperability between diverse platforms. Moreover, open-source frameworks encourage continuous improvement through community-driven contributions, allowing for rapid adaptation to emerging challenges and technological advancements. As Industry 4.0 relies on seamless data exchange and digital integration, open-source solutions serve as a catalyst for scalability, flexibility, and adoption of AAS ecosystems. In this context, there are several studies [3,18,19,20,21] that have tested and provided preliminary information about open-source implementations, comparing them according to different criteria. Each offers distinct features, integration capabilities, and technological approaches. These findings put forward valuable insights, providing a comprehensive understanding of the strengths and limitations of existing AAS implementations. However, with the growing number of open-source solutions, assessing their practical applicability is limited in industrial environments. For that reason, we further compared open-source AAS solutions to realize the potential of AAS open-source tools within the Industry 4.0 ecosystem.

Given the evolving nature of Industry 4.0 and its rapidly advancing technological landscape, the methods and models used in these studies are expected to change over time. These variations stem from the continuous involvement of different participants in the Industry 4.0 platform, integrating new elements into development processes and creating prototypes with increasingly comprehensive and innovative functionalities. As an illustration, in a recent article [3], open-source implementations were compared with each other in terms of interface, programming language, communication protocols, etc. Since then, these applications have continued to be developed, and new versions have been released. However, the literature review in the paper identified four main active applications, namely AASX server, Eclipse BaSyx, FA³ST service, and NOVAAS. When our review was conducted at this time, it again showed that other open-source applications had still not been active for a long time or were under development, so we focused on these four main applications.

This study aims to share development experiences in real settings as well as information that can be accessed related to the tools. The purpose of the study is not to make generalizations but to contribute to research in the form of an empirical work. In this paper, four open-source AASX implementations, AASX server, Eclipse BaSyx, FA3ST service, and NOVAAS, are herein reviewed. Our study evaluates their different multiple characteristics using both simple models and the real assets used in a petro-chemical facility.

## 2. Materials and Method

In this section, elementary aspects related to Asset Administration Shell are presented in Section 2.1, Section 2.2, Section 2.3 and Section 2.4. Section 2.1 introduces the AAS metamodel, describing its role in consistent modeling. Section 2.2 focuses on data specification, emphasizing templates that enrich the metamodel with additional semantic attributes and demonstrating instances for standardized representation of asset information with use cases in data exchange. Section 2.3 describes submodel descriptions, highlighting standardized templates approved by IDTA, which facilitate effective digital twin creation and management. Eventually, Section 2.4 discusses semantic versioning, explaining its significance in maintaining standardized and interoperable AAS content in the Industrie 4.0 ecosystem.

GitHub repository [22] contains the AASX file that was built for modeling industrial columns in this study and explains how to use open-source implementations together with the installation procedure.

To comply with security requirements set by the petrochemical company, physical assets’ model elements were anonymized by renaming, and the real data collected from the assets and the sensors installed on these assets were excluded from the repository. Details on the case study are available in the Case Study Section of this article.

### 2.1. AAS Metamodel

A metamodel is an abstract framework that defines the structure, semantics, and constraints of a specific domain model. It serves as a blueprint for creating models within that domain, ensuring consistency and standardization. In software engineering and systems design, metamodels provide the rules and guidelines for model creation, enabling interoperability and understanding across different systems and stakeholders. Since the beginning of the AAS work, there have been changes in the metamodel work, which has resulted in a progressive change in version numbers. The transition from V2.0.1 to V3.0RC02 represents a significant evolution of the AAS metamodel [23], characterized by Table 1.

### 2.2. Data Specification

Data specification templates within the AAS define the additional attributes required for different element instances beyond the core metamodel. Each template is tailored to a specific scope, such as concept descriptions or operations, allowing for specialized semantic enhancements. An element instance can utilize multiple data specification templates through the HasDataSpecification relationship, providing the flexibility to meet complex data requirements. Instead of depending solely on external global references, embedded data specifications are composed of pairs that include an external global reference to a data specification along with the embedded content of that specification within the AAS. This dual approach ensures that the AAS can reference standardized data specifications externally while also maintaining self-contained definitions, thereby enhancing both robustness and flexibility. Currently, the primary template supporting IEC 61360 within the AAS is DataSpecificationIec61360, which defines concept descriptions for both properties and coded values in alignment with the IEC 61360 standard [24].

**Figure 1:**
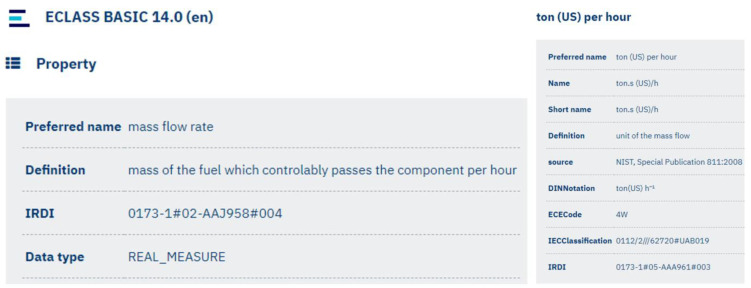
ECLASS Definition of Mass Flow Rate [25].

**Figure 2:**
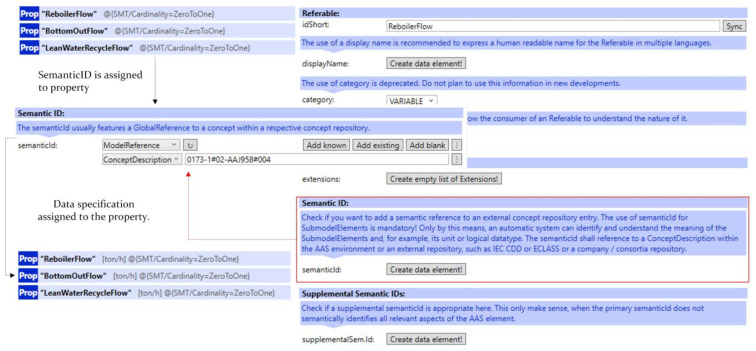
Concept description Assignment to Properties.

**Figure A1:**
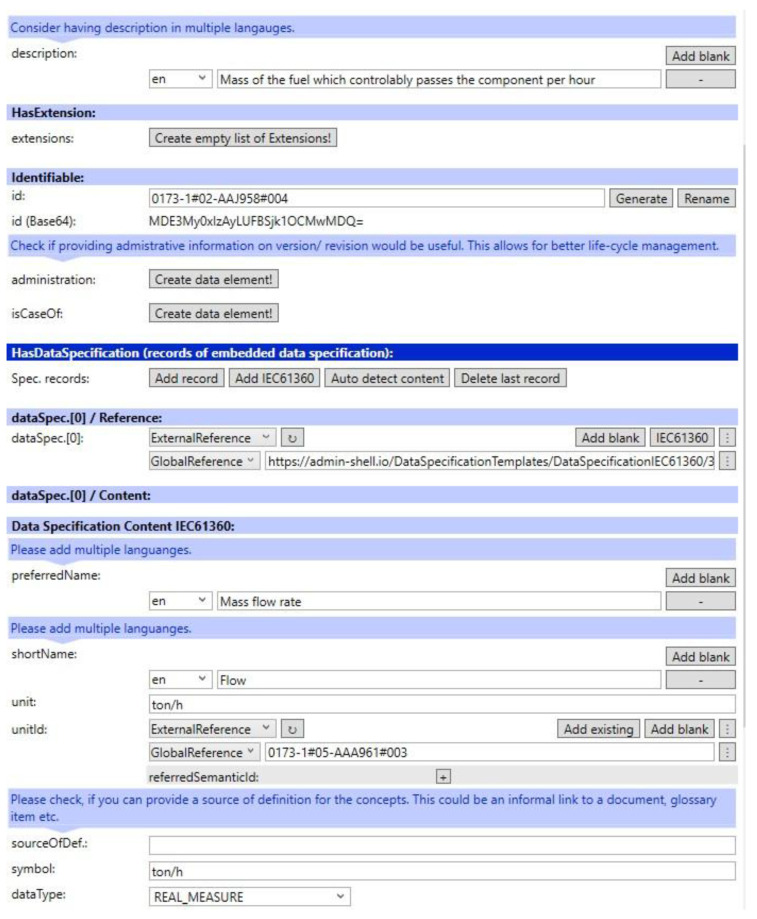
Generated Concept Description of Mass Flow Rate in AASX Package Explorer.

Submodel templates approved by IDTA already include predefined specifications within their submodel elements. However, since the sensor metrics used in this study did not exist previously, ConceptDescriptions for these metrics were defined using the AASX Package Explorer. During these additions, property definitions and corresponding data types specified under the IEC 61360 standard were used to ensure consistency and interoperability. In Figure 1, the definition of mass flow rate and its unit, ton/h, is illustrated, which belongs to ECLASS version 14.0. Accordingly, Figure 2 shows how these definitions are represented in the AASX Package Explorer interface. The assigned ConceptDescription details can be found in Figure A1 in Appendix A.

The same ConceptDescription can be used in multiple properties that share a common measurement metric. When a ConceptDescription is referred to a property, the derived data specifications will be assigned to this property.

**Figure 3:**
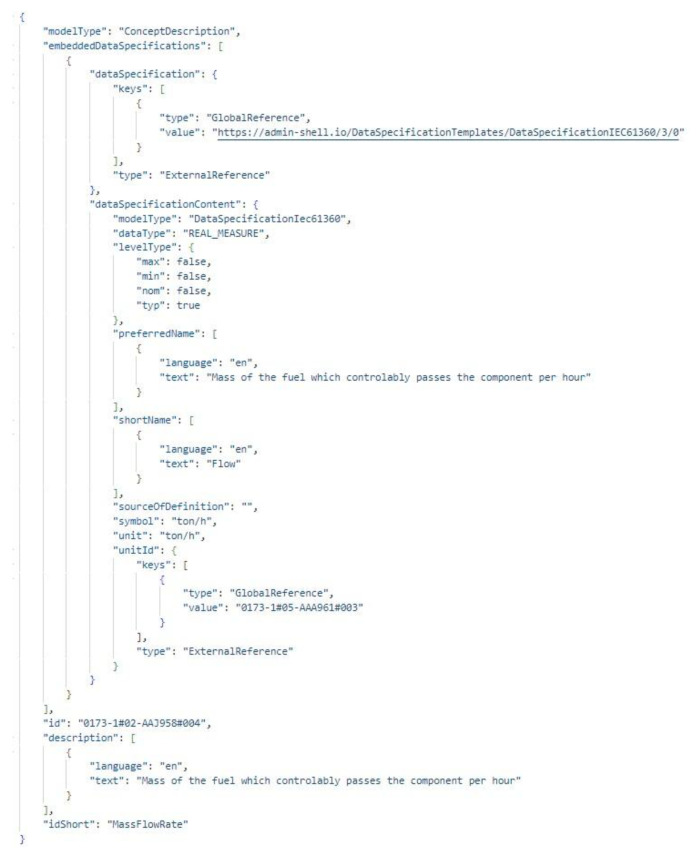
Received Embedded Data Specification of the Mass Flow Rate Property in JSON.

To enable efficient data exchange and integration, the AAS supports multiple serialization formats, with JSON (JavaScript Object Notation) being a primary option. Additionally, JSON is utilized to define payloads in HTTP/REST APIs, which are essential for active AAS instances that participate in real-time communication and data exchange within networked environments. Since the ID of ConceptDescription is unique and base-64 encoded as well, when property definitions are to be obtained, it is sufficient to make a request to the identifiable address of the ConceptDescription. This will return an embedded data specification that is assigned to the property, as shown in Figure 3.

**Figure 4:**
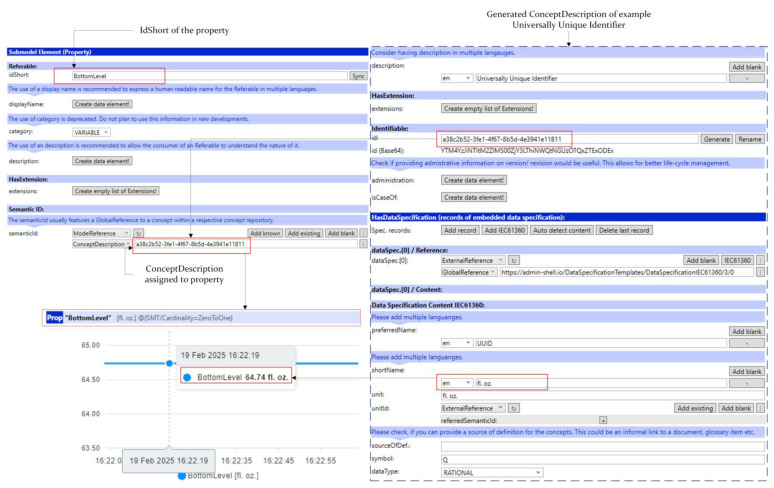
Example Definition of a Custom Identifier.

If data types or concept definitions that the user wants to define are not included in the datasets or are used inhouse, users can still define the data types they want as an embedded data specification. Figure 4 below shows an example of a use case for the Universal Unique Identifier (UUID), a 128-bit identifier.

### 2.3. Submodel Descriptions

The submodels used in AAS files are approved by IDTA, and they can be found in the GitHub repository, consisting of several submodel templates [26]. The repository has multiple versions of submodel templates provided by different developer groups. Although the created submodel templates basically use the same standard semantic identifiers, they are created to serve different underlying purposes and have different characteristics depending on the manufacturer. Therefore, the repository of each submodel template contains the scope, version, compatibility, and status of the designed submodel, and each template indicates it is supported by AASX Package Explorer [27]. In our submodel selection, we checked that the semantic version of the submodel is supported by the BaSyx plugin; this allows us to visualize the digital nameplate and technical data of the assets for which we want to create a digital twin and shows the manufacturer’s information and basic product specifications. The templates we found suitable for these selections had more than one version type, and some applications received a 409 conflict response on the BaSyx server, which is an error message caused by an incompatibility between the user’s request and a previously specified rule or version difference on the web server. Thus, we used the versions with no conflict and described them, given in Section 2.3.1, Section 2.3.2 and Section 2.3.3.

#### 2.3.1. Digital Nameplate Submodel

The Digital Nameplate Submodel template [28] aims to provide asset nameplate information to the Asset Administration Shells in an interoperable manner. The central element of this template is the provision of properties that can be defined using dictionaries such as ECLASS and IEC CDD. In the current version, an IRI is provided as a semantic identifier for some of the specified properties. The purpose of this document is to present submodel definitions that enable the meaningful exchange of information about assets and their nameplates among partners in a value creation network. Standardized metadata have been defined for equipment used in the process industry and factory automation [29].

#### 2.3.2. Technical Data Submodel

The Technical Data Submodel template [30] facilitates the standardized sharing of asset technical data within an Asset Administration Shell. It uses properties defined by interoperable dictionaries like ECLASS and IEC CDD as well. The main goal is for manufacturers or suppliers to describe assets in a way that system integrators or operators can easily understand. This submodel consists of four areas: general information for basic details about the equipment and provider to support identification and ordering; product classification for categorizing the asset as a commercial product; technical properties for detailed technical data, organized and identified by semanticIds; and further information for additional manufacturer details and validity dates [31].

#### 2.3.3. Time Series Submodel

The Time Series Submodel [32] represents an approach for the semantic description of time series throughout the asset lifecycle within the Asset Administration Shell. Given the availability of many database systems optimized for time series data, this specification defines the integration of external data sources as well as the storage of time series data directly within the AAS. Additionally, it offers an operational perspective to enable standardized queries, inputs, and functions on time series data. It supports the integration of time series data within the AAS or from external sources, covering use cases with various storage options for data from physical and virtual sensors [33].

Time series can be divided into three primary logical segments, which can be labeled and described with additional semantic information:

- InternalSegments: Allows the management of the time series data structure and content directly within the AAS, for example, pre-adding data records as Submodel Collections during the creation of an AASX file;
- LinkedSegments: Enables the client to access an endpoint and query an external system to manage the time series in real time outside the AAS;
- ExternalSegments: Involves embedding a data file containing time series data with different formats into the AASX file.

In order to enable real-time tracking of values within the digital twin, the LinkedSegments section is utilized. This allows data to be retrieved as Submodel Element Collections by entering the desired query parameters on the specified endpoint. The IDs of the data to be fetched must be uniquely defined in the Record section located in the metadata of the submodel.

**Figure 5:**
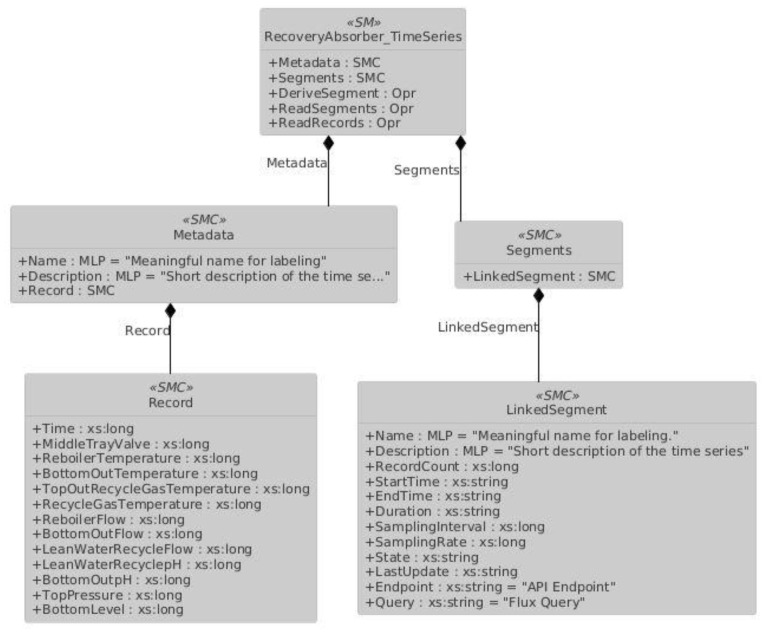
Partial UML Diagram of Time Series Submodel.

Below, in Figure 5, the LinkedSegment section for the RecoveryAbsorber unit is partially provided. The Record and LinkedSegment IDs presented here are semantically recognized by the Eclipse BaSyx processor, which can then send requests accordingly.

### 2.4. Semantic Versioning

One of the key features of Industrie 4.0 is the creation of a unified workspace and vocabulary for all platforms, standardizing data serialization and product identification. In this context, the structure and syntax of Asset Administration Shells (AAS) are defined by IDTA [34]. Additionally, semantically defined content, along with its characteristics, qualities, and values, is also required.

Currently, there is no single, unified vocabulary for semantic identifier definitions. Therefore, alongside identifiers defined by admin-shell-io [35], external common data dictionaries such as ECLASS [25] and IEC CDD (Common Data Dictionary) [36] can also be used. Although these dictionaries contain definitions for various data types and assets, to facilitate the rapid creation of specific identifiers, IDTA has developed a self-registering identifiers repository [37]. For identifiers obtained from these sources, IDTA has defined internationally accepted identification types suitable for use in AAS, such as IRDI (International Registration Data Identifier) and IRI (Internationalized Resource Identifier) [23].

Additionally, for companies that operate in an in-house manner, custom identifiers are offered as a third option. By applying semantic mapping strategies, it is possible to ensure interoperability between systems that use IRDI data from different dictionaries or ontologies. Manufacturer-specific information and functions can be represented and communicated using IRIs, URIs, URLs, and internal custom identifiers, similar to standardized information and functions. Currently, some identifier descriptions are being standardized by IDTA using the following URI pattern: http(s)://admin-shell.io/<sub-namespace>[/<version>[/<revision>]]/<ShortId>[/<AttributeShortId>[/<ValueShortId>]].

Using this URI pattern, it is possible to register definitions for standardized or manufacturer-specific assets or modules such as submodels and their components. The registration process has a standard defined by IDTA as well. The semantic-based workflow is suitable for complex subjects where existing semantic definitions are used, or new semantic definitions are created. The working team first develops the design approach for the submodel template elements and divides them into submodels, and then, these definitions are transferred to the AASX model. As the semantic definitions mature, AASX files are created manually or through automated methods and verified through internal reviews. Subsequently, the submodel template specification document is prepared and submitted for official review. In the final stage, both AASX files and semantic models are published, ensuring that submodel templates are created in a systematic and collaborative manner. This process includes important steps to maintain semantic integrity and enhance reusability [38]. While many of these definitions are still under development, the IDTA-approved submodel templates can be accessed via the content hub [39] and the corresponding GitHub repository [40]. In the asset model built in this study, care was taken to use approved identifiers for the submodels. For example, Time Series Submodel uses the identifier “https://admin-shell.io/idta/TimeSeries/1/1”. The use of this identifier can be used to trigger plugin mechanisms in BaSyx. If the semanticId that identifies the submodels is found in BaSyx’s defined plugin list [41], a visual interface compatible with the semanticId is presented in the visualization view of the UI. As an illustration, for the Time Series submodel, it is a graphical interface with various types of charts, while for the digital nameplate, it is an interface that visualizes the contact details of the product, the logo, and the address of the manufacturer. Although the current visualization panel is available for a limited number of semanticIds, externally developed custom plugins can also be integrated into BaSyx.

**Figure 6:**
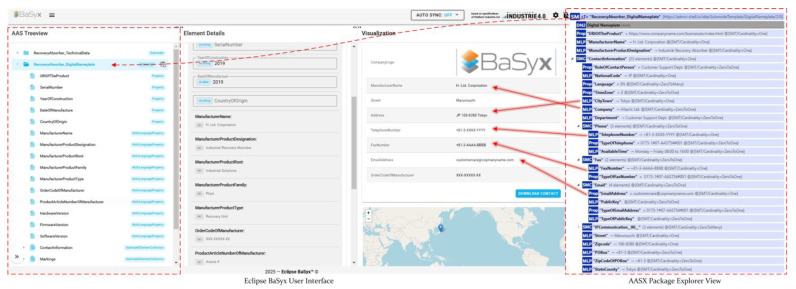
Digital Nameplate Visualization of Recovery Absorber Asset in BaSyx.

Below, the plugin visualization of the semanticId “https://admin-shell.io/idta/SubmodelTemplate/DigitalNameplate/2/0” that read from the created AASX file is given. For the parts shown with arrows in Figure 6, the AAS treeview panel on the far left reads and visualizes the AAS file uploaded to the system, while the visualization part on the right presents the predefined IdShorts semantically parsed by the Eclipse BaSyx backend as a visual format. At the same time, it can transfer the semantically unified parts into manufacturer’s location in 2D. In addition to the visualization, there is also a function that exports manufacturer information as contact info. Once the digital nameplate is detected, the plugin feature can detect IdShorts such as ContactPerson, Department, Address, and Phone in the sub-elements of the submodel and convert them into contact cards. In this respect, it is important that IdShorts are used in the structures published by the IDTA, which is also important for establishing a general order in the digitalization process.

## 3. Results

### 3.1. Evaluation Approach

Research into AAS tools and technologies is aimed at addressing these core issues to enable widespread adoption and ensure that AAS becomes a cornerstone of digital transformation in industries. The development and implementation of AAS tools and technologies face several core issues. These issues include interoperability across platforms, data integration and management, security and privacy concerns, scalability and real-time performance, complexity in modeling and maintenance, data ownership and governance, life-cycle management and asset sustainability, and adaption and implementation challenges. This study addresses the adoption and implementation challenge of AAS technology because the adoption of AAS technologies in industries is still relatively slow due to factors such as high implementation costs, lack of expertise, and resistance to change. This slow adoption results in small- and medium-sized enterprises (SMEs) to face challenges in implementing AAS due to resource constraints. Aiming at helping SMEs willing to apply AAS, this study compared different solutions and presents the obtained results.

In this evaluation, we began by noting that the AAS metamodel V2 is no longer maintained, so metamodel V3 became the primary focus for creating and testing our AAS files. As of now, there is no officially standardized Industrie 4.0 language available for Type 3 AAS [19]; consequently, platforms were not adopted for Type 3 AAS implementations. As Type 3 AAS is still in the early research phase, Type 2 remains the best practical solution for integration with sensors, devices, and production systems that reveal real-time information about event-driven updates on product status. Therefore, the examinations were carried out according to Type 2 implementations. We then defined a set of evaluation criteria, encompassing each platform’s ease of deployment, metamodel compliance, real-time data monitoring, user interface capabilities, and overall suitability for industrial use cases. The assessment process involved two principal stages:

1. Documentation Review and Theoretical Assessment:

Our study conducted an in-depth reading of user guides, review papers, developer wikis, and related documentation to map each platform’s stated capabilities against our criteria. The aim was to comprehensively map each platform’s declared capabilities against our predefined evaluation criteria. Although we primarily relied on existing documentation and source code to assess most of these criteria, we also performed deployment tests to verify which AAS metamodel elements are fully supported and how the implementations are handled. Although there are studies on similar software packages, which were introduced in the Introduction Section, addressing different aspects of the tools and evaluating them with different criteria, we present in the Results Section the criteria not mentioned in these studies, which were updated after the date of the study and which we deemed necessary for the case study we conducted. Based on our combined findings, we aggregated the data into Table 2 and Table 3 as comparison tables to highlight strengths, weaknesses, and unique differentiators.

2. Practical Deployment and Performance Testing:

In this empirical evaluation stage, an experimental test scenario was developed to quantitatively analyze each implementation’s resource utilization and performance. A sample AASX file with metamodel V3 served as the input for BaSyx, FA^3^ST, and AASX server and metamodel V2 as an input for NOVAAS. For practical deployment, Docker containers were built from official repositories provided by each respective implementation. Next, the resource consumption profiles and differences in efficiency under comparable workloads are summarized in Table 4.

**Figure A2:**
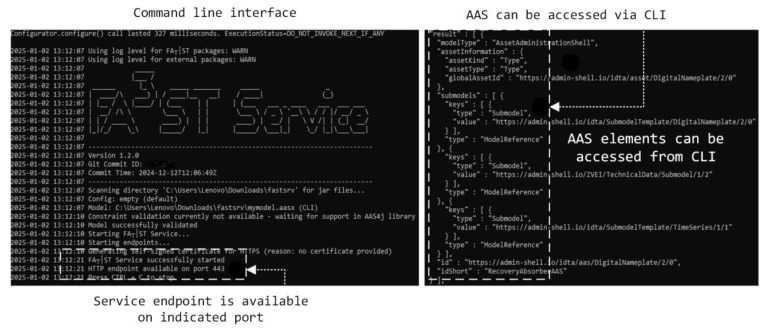
FA^3^ST Service CLI.

**Figure A3:**
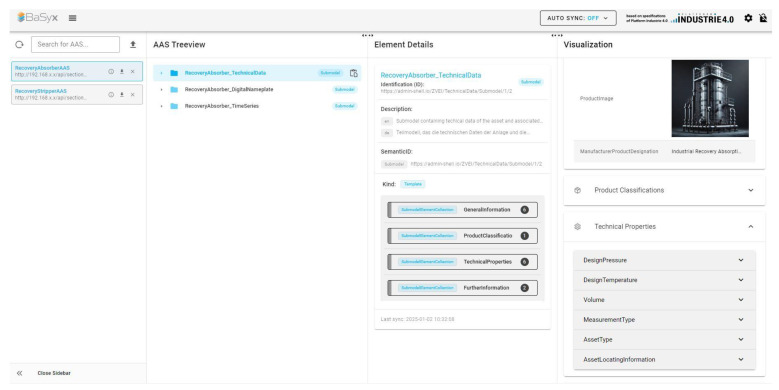
Eclipse BaSyx User Interface.

**Figure A4:**
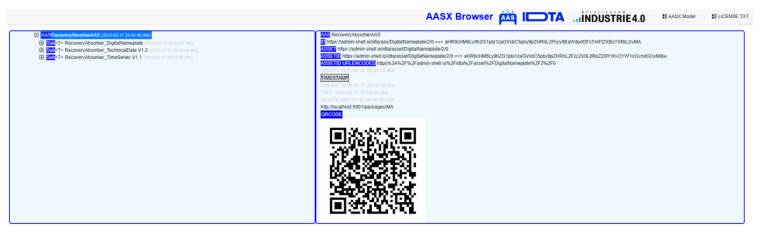
AASX Server User Interface.

**Figure A5:**
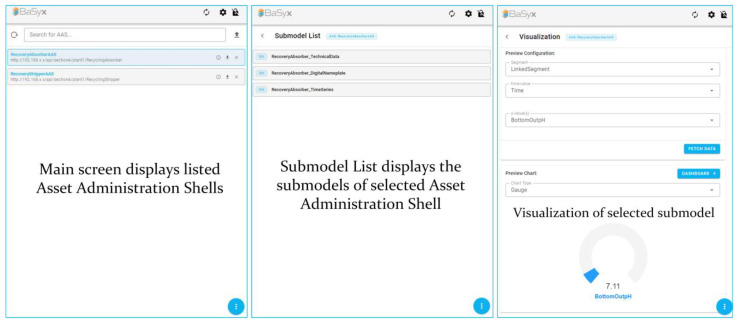
Sequential View of Eclipse BaSyx Mobile Menus.

While testing existing open-source implementations, the AASX file was created using the AASX Package Explorer to ensure it conformed to the specified structure. The version used was v2024-06-10.alpha [42], which supports metamodel V3 format for AAS provisioning. The referenced GitHub repository contains multiple versions of the AASX Package Explorer, with some versions supporting only metamodel V3 and others supporting only metamodel V2. There is no version that supports both V2 and V3 simultaneously, and this limitation also applies to open-source implementations. Due to differences in serialization, open-source implementations provide separate models for V2 and V3 compatibility. Thus, the versions that comply with the metamodel were used. Out of the four open-source tools evaluated, three offer full support for metamodel V3: AASX server [43], FA^3^ST service [44], and Eclipse BaSyx [45]. NOVAAS offers partial support, with ongoing development indicated for future updates [46]. A generated AASX file can only be properly displayed by open-source implementations that fully support metamodel V3. FA^3^ST does not have a graphical user interface. Among the technologies we compared, the user interface of Eclipse BaSyx is the most user-friendly and easy to use. The user interface samples of each technology are shown in Figure A2, Figure A3 and Figure A4 in the Appendix A. BaSyx additionally offers an interface for mobile support similar to the desktop version described in Figure A5, where the page flow is from left to right in order to visualize time series data.

### 3.2. Evaluation Results

The integration and usability aspects of Table 2 and Table 3 for the reactive AAS implementations reveal a balance between ease of deployment and the degree of configurability. Generally, systems that prioritize a simple setup tend to be more accessible for end users and engineers alike. Such systems typically offer integrated visual interfaces that facilitate live editing and rapid prototyping, thereby reducing the barrier to entry for non-programmers. In contrast, solutions that emphasize extensibility and flexibility provide a richer set of configuration options through multiple files or environment variables and are often supported by comprehensive SDKs. These systems, however, require a steeper learning curve and deeper technical understanding, particularly when integrating with external registries, enforcing security policies, or customizing asset synchronization. Overall, while some architectures like AASX server and NOVAAS offer a streamlined and user-friendly experience with minimal coding requirements, Eclipse BaSyx and FA^3^ST service deliver enhanced configurability and extensibility at the cost of increased complexity. This trade-off is essential to consider in the context of the intended application domain, whether it is for rapid prototyping or for scalable, secure industrial deployments.

Table 4 shows the deployment tests and results for four different open-source implementations. For the metrics in the columns, build time represents the time it takes to build applications on Docker from scratch using composed files. Image size is the disk space occupied by the docker image, start-up time is the time it takes for the containers to go from off state to healthy state, and idle memory and CPU usages are the usage when the applications are idle, that is, when they are not receiving any requests. As a test scenario, a total of 400 GET requests were sent periodically in 20 s to obtain the description of the single AAS file integrated into the applications and CPU and memory usage of containers. Looking at the results, it can be observed that the AASX server in particular has a low resource consumption. Additionally, FA³ST service and NOVAAS also have low resource consumption values. BaSyx, on the other hand, requires relatively better hardware in terms of CPU and memory. Based on this, it can be said that the four different implementations can serve different types of purposes. For example, an application that can efficiently utilize limited resources can be chosen for a lightweight asset, whereas a powerful hardware such as a PC can be used for applications that use more resources and therefore offer extra features, as shown in Table 2.

In addition to the properties in Table 2 and Table 3, in order to test the applicability and extensibility for the digital twins of the industrial recovery columns on time series, the following reasons contributed to the selection of BaSyx to perform the case study:

1. Deprecation of AAS Metamodel V2: Since metamodel V2 has deprecated, and further developments are focused on metamodel V3, the AAS file for this study was generated with the metamodel V3 structure. While the other three applications have versions compatible with metamodel V3, NOVAAS has a current version fully compatible with metamodel V2. There is also another version with limited sub-element support for metamodel V3, which might be extended to full support in the future, but since the other sub-elements in the AAS file we created that are not yet supported by NOVAAS caused reading errors in the file, the focus was shifted to the other three implementations;
2. Monitoring Capabilities: For the three selected software options, monitoring capabilities are critical in industrial applications. Hence, preference was given to software with a built-in graphical interface, excluding FA^3^ST service, which currently operates via a CLI (Command-Line Interface);
3. Visual Interface: While the AASX server primarily displays data in a list format, Eclipse BaSyx provides submodel-specific visualizations by utilizing semanticIds within submodels. This enables better recognition and understanding of the submodel’s details;
4. Time Series Data Monitoring: Eclipse BaSyx offers a graphical interface for Time Series Submodels through its semantic visualization feature, enabling effective data monitoring;
5. Mobile Platform Support: In industrial environments, access to a computer is not always feasible. Eclipse BaSyx is the only solution that provides mobile platform support, addressing this need;
6. Comprehensive Documentation: Eclipse BaSyx offers detailed documentation for both users and developers. Additionally, GitHub repositories provide example use cases for various integrated end-user applications and protocol integrations, significantly speeding up and simplifying the creation of different test scenarios;
7. Eclipse BaSyx supports real-time AASX import and export functionalities and offers an AAS editor tool within its UI. This allows visual modifications to AAS elements, making Eclipse BaSyx a useful tool for partial AAS editing as well.

## 4. Case Study

### Asset Administration Shell

**Figure 7:**
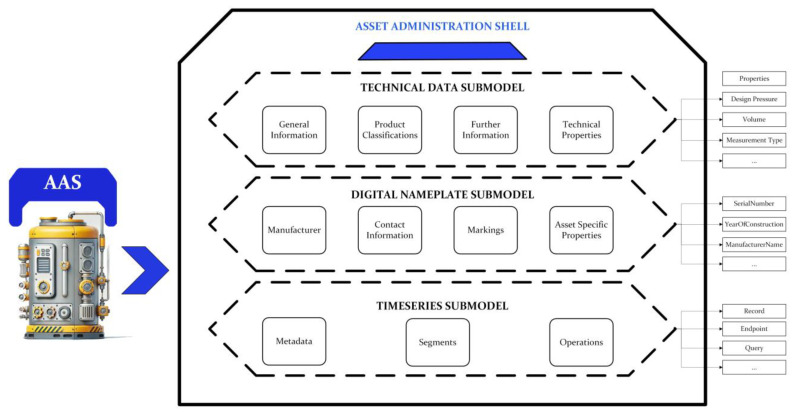
Generated Asset Administration Shell Structure of Industrial Asset.

**Figure 8:**
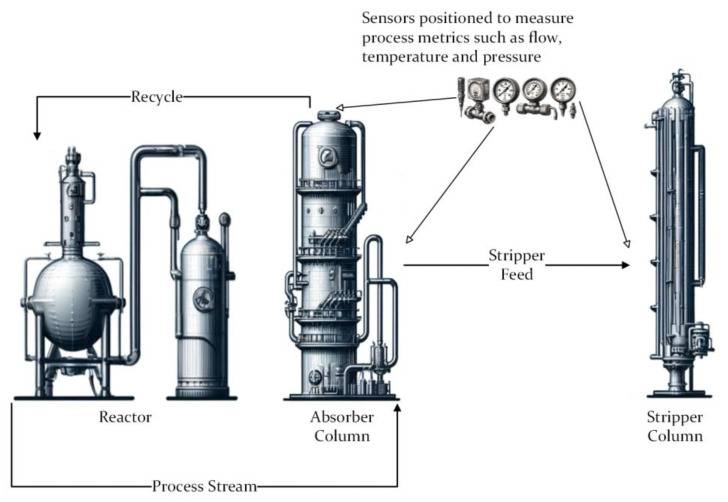
Process Flow of Recovery Units.

The structure of the prepared AASX file is illustrated in Figure 7, which represents the digital twin of the ethylene oxide/ethylene glycol (EO/EG) plant EO recovery unit columns. The recovery absorption unit is a system used to capture and recover EO vapors from the gas stream using a liquid absorbent. The recovery stripping unit is a process unit used to recover EO absorbed in the liquid phase by heating and vaporizing it into the gas phase. These two systems are connected as described in Figure 8.

To ensure the proper operation of the process, sensors’ measuring metrics, such as flow, pressure, temperature, and pH, are positioned at various points in the columns. Since the goal of this study is the process monitoring of a digital twin, detailed technical data for the sensors were not created. Instead, the Time Series Submodel includes measurement metrics and units within its metadata. To facilitate better identification of the columns and access to contact information, a Digital Nameplate Submodel was included. Additionally, a Technical Data Submodel provides access to technical details. Since both columns have similar structures, they were created using the same kind of submodels by modifying submodel elements.

**Figure 9:**
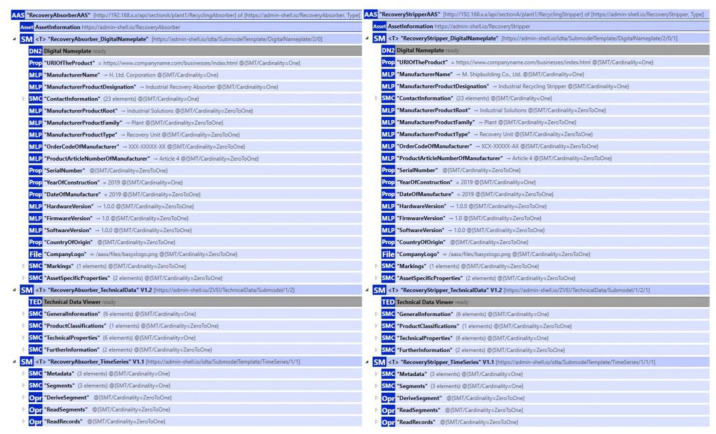
AASX Package Explorer Views of Column Assets.

For process monitoring, the need to examine both assets together led to integrating them within the same AASX file. V2024-06-10.alpha release of the AASX Package Explorer [42] allows the use of different submodels that share the same semanticId by generating a base64-encoded submodel identifier for each submodel. This ensures each submodel remains unique, preventing conflicts with other submodels. Since Eclipse BaSyx is fully compliant with AAS metamodel V3, no problems were encountered while using the AASX file shown in Figure 9.

Since EO recovery unit columns operate in real life and are managed via a closed control system, historical data associated with the system were collected in the form of sensor tags and measurements, and the case study was conducted based on these data.

**Figure 10:**
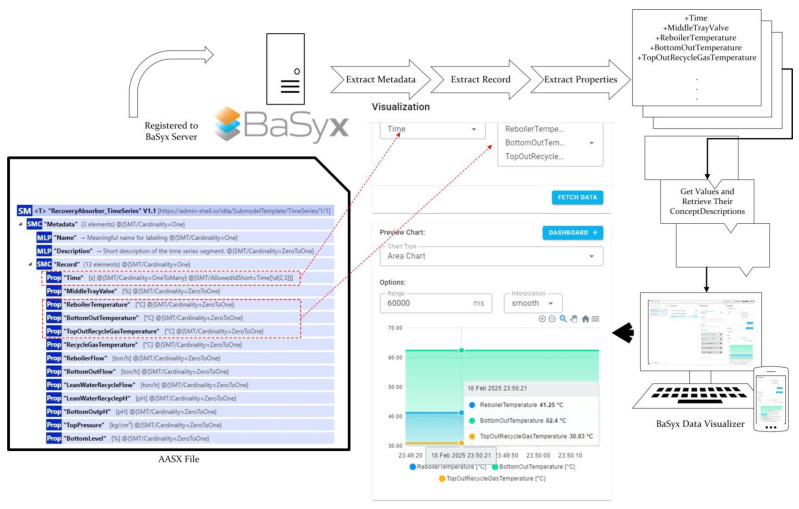
Eclipse BaSyx Visualization of Time Series Submodel.

The collected data topics were classified into two categories based on the type of industrial column and are further subdivided according to the types of connected sensors. During data simulation, data are published via an MQTT broker, tagged with the column type and sensorId. The Eclipse BaSyx backend processor uses data mapping to access this tagged data by parsing the AASX file and obtains the stored data within the Time Series Submodel. This also leads to the identification of the submodel through the semantic definitions described in previous sections. BaSyx creates a visualization segment specific to the submodel. It then agglomerates the properties in the Record section within the metadata and retrieves their respective ConceptDescriptions. This approach, which is illustrated in Figure 10, ensures that the variables in the graph are accurately visualized with their units, facilitating clear and accurate representation of the time series data.

Regarding the system’s general operation principle, the Time Series Data Docker file example from BaSyx’s GitHub repository [47] was used as the backbone. The Docker containers were modified to send historical data for two different column types to the MQTT Node via two separate MQTT clients, each tagged with the current timestamp.

**Figure 11:**
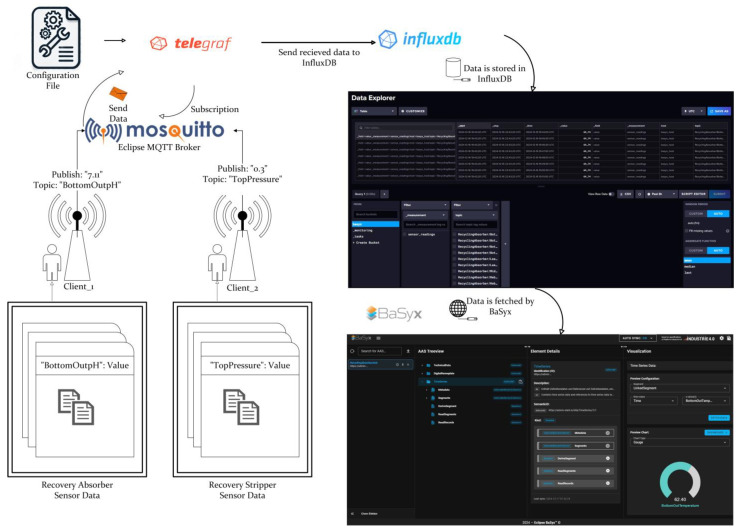
System Flow Diagram.

**Figure 12:**
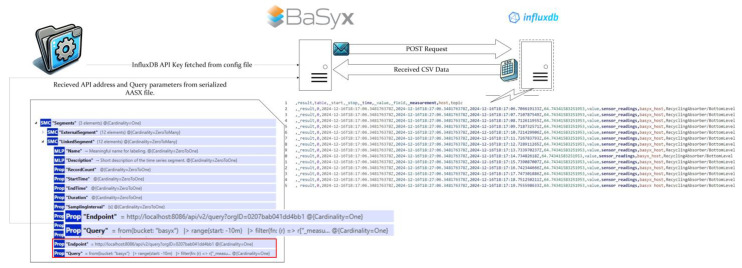
Data Transfer from InfluxDB.

**Figure 13:**
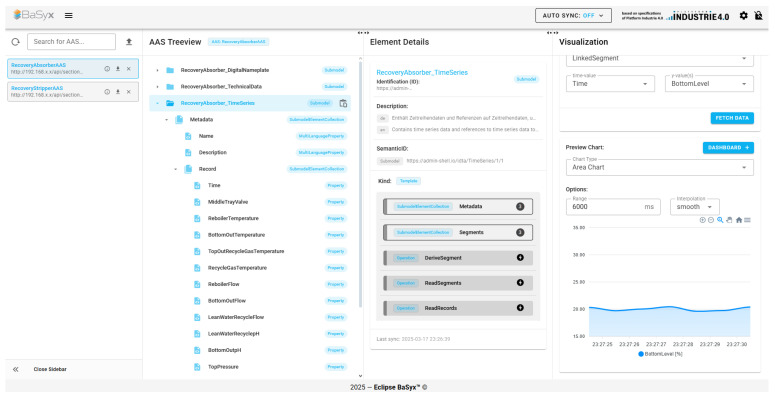
Displaying the Received Data on the User Interface.

The data collected on different topics in the MQTT Node are forwarded to InfluxDB via a Telegraf container. In InfluxDB, the data are stored, allowing historical data to be examined over specific time ranges. When BaSyx needs to access the data stored in InfluxDB, an API request is sent. This request requires information such as the data location in InfluxDB and the endpoint to which the request will be sent. These details are obtained from the Endpoint and Query parameters within the LinkedSegments structure defined in the AASX file. When the API request is successful, a CSV file is returned containing the start time, stop time, value, and topic. This CSV is processed by the BaSyx backend and presented in a visual format. As shown in Figure 11, the sensor readings are sent to the MQTT node via two different clients: Telegraf, which is constantly subscribed to this node, sends the published data instantaneously to InfluxDB, where the data are stored with timestamps. This data stored in InfluxDB are transferred from the database to the BaSyx interface via the request sent by Eclipse BaSyx, as shown in Figure 12. BaSyx takes the parameters such as Endpoint and Query from where they are highlighted in AASX file and the API key from the environment file in the Docker build, then merges them in the sent request. The resulting data are visualized according to the inputs entered in the visualization panel shown on the right in Figure 13.

Besides UI, BaSyx supports application programming interfaces defined by IDTA [48]. The API employs standard HTTP methods, such as GET for retrieving resources, POST for creating them, PUT for replacing them, PATCH for partial updates, and DELETE for removing them, ensuring compatibility with widely adopted REST principles. It supports IdShortPath for structured addressing of submodel elements and Base64Url encoding for globally unique resource identifiers. The API enables online access to entities and submodels and provides important features for large-scale applications such as pagination, serialization modifiers, and dynamic updates. With Swagger UI and OpenAPI integrations, developers can easily explore and test API endpoints.

## 5. Discussion

Among the tested open-source implementations, Eclipse BaSyx was determined to be the most suitable application for creating a digital twin of the industrial recovery columns. The reasons for this decision include its robust middleware architecture and extensive plugin-driven extensibility, offering significant flexibility for diverse integration scenarios. Moreover, Eclipse BaSyx distinguishes itself as a middleware architecture, which enables flexible integration layer that simplifies connecting heterogeneous data sources and services. Its architecture is notably extensible through plugins, allowing seamless addition or adaptation of functionalities and integration patterns without significant changes to the core system. This plugin-driven approach enhances adaptability and reduces the effort required for extending and customizing the system according to specific industrial use cases. Other open-source solutions, on the other hand, can serve different cases of industrial purposes. For instance, FA³ST uses an edge-synchronized design, and it focuses on more real-time synchronization at the edge. When minimal latency is needed, this approach can be very useful. As another example, AASX server can be integrated on cost-effective assets since it operates with minimum resource consumption, or NOVAAS can be preferred for the configurable event-driven architecture it offers to the user. In short, it would not be correct to conclude that a particular AAS tool is the best option among the available open-source AAS solutions, but it can be said that the diversity of open-source solutions increases the usability for different purposes and facilitates integration into many different devices and platforms due to the fact that they are built on different architectures.

When viewed through the lens of recognized AAS categories, it becomes clear that most solutions are limited to passive (providing static metadata) or reactive (responding in real time to external events) functionalities. AASX server, for instance, facilitates a primarily passive approach that is well suited for rudimentary or demonstrative scenarios where metadata hosting suffices. By contrast, Eclipse BaSyx and FA³ST service exhibit reactive features, affording richer event-driven responsiveness and interoperability with various protocols, yet none fully embraces the proactive dimension—namely the ability to learn, predict, and autonomously adapt within complex industrial environments.

To move beyond these passive and reactive solutions, it is important to underscore the need to develop proactive AAS implementations that function not merely as “data containers” but rather as “cognitive engines”. Realizing such engines will entail tackling three key bottlenecks, namely ensuring low-latency interoperability across diverse industrial protocols and IT systems, enabling meaningful collaboration among heterogeneous networks of machines and services, and embedding advanced analytics such as federated learning, causal inference, and self-adaptive decision making directly into the AAS architecture. Achieving these capabilities will push the boundaries of digital twin technology toward genuinely autonomous operation, marking a fundamental shift from reactive data processing to predictive, self-optimizing behaviors.

## 6. Conclusions

In the present study, four open sources were reviewed. As a result of this review and for the reasons listed in the article, it was decided that BaSyx is the middleware that can work most efficiently with our AASX file, which is the digital representation of our industrial assets. Our study tested Type 2, i.e., the reactive Asset Administration Shell, on BaSyx with the data we published on the MQTT node. This allows the variables on the AAS file to be changed or read instantaneously via endpoints. These variables also belonged to a real industrial recovery column, so, in fact, our theoretical work can be applied to real operating industrial equipment operated in real time. The only difference here in the simulation is that the industrial assets are already in operation, and the data are read via cable and written to the central control system with various automation protocols, whereas in the presented project, these data were converted into a virtual MQTT stream. However, BaSyx can connect to various automation protocols using a databridge. For instance, there are plugins such as OPC2AAS [49] for OPC-UA, one of the most widely used protocols. This means that if open-source implementations continue to be developed, it can greatly accelerate the digitalization process within the scope of Industry 4.0 with its further standardized AAS structure. Also, various submodel templates are still being developed, and more advanced digital twins with different plugin features may be seen in the future. The creation of digital twins enables the monitoring of real assets’ characteristics, such as lifecycle and health. Thus, when deep learning or machine learning models are integrated into the digital twin with the collected data, inferences can be made, such as failures that may occur on the asset and the maintenance work that needs to be planned. In addition, scenarios that can be applied to increase efficiency can be tested.

On the other hand, when looking at the factory floor today, we often find SCADA systems, PLCs, etc., using protocols like OPC UA, MQTT, or fieldbuses. AAS implementations are relatively new entrants. They can complement existing systems: for instance, an AAS could be layered on top of a PLC’s OPC UA server to provide standardized access and combine metadata with live data. Over time, as more devices natively provide an AAS, or more factories adopt AAS frameworks, we may see them taking over roles that custom OPC UA information models or proprietary historians play now. Traditional IoT solutions are currently more feature-rich in specialized areas (e.g., a cloud IoT platform might have built-in machine learning for predictive maintenance). The AAS ecosystem would integrate with such services but is not there to replace analytic tools—it is there to ensure the data feeding those tools are well structured and interoperable.

In future versions of this study, we will work on the feasibility of these scenarios and the feedback that may be reflected in the system.

## Figures and Tables

**Table 1:** Evolution of AAS Metamodel from V2.0.1 to V3.0RC02 [23].

Aspect	Description
Higher Flexibility	Revised asset representations improve interoperability and ensure that the metamodel is compatible with global standards.
Robust Formalization	Comprehensive constraints and formal grammar definitions provide data integrity and consistency and facilitate reliable implementations across diverse applications.
Simplification	The removal of deprecated and unused elements reduces complexity, making the metamodel more focused and easier to implement.
Future Enhancements	The introduction of experimental features paves the way for continuous development and innovation in the AAS ecosystem.
Data Specification	Aligning data specifications with IEC61360 and introducing new data types allows many data integrity issues to be included in the AAS methodology.

**Table 2:** Feature Comparisons of Open-Source AAS Implementations.

Criteria/Specs	AASX Server	Eclipse BaSyx	FA^3^ST Service	NOVAAS
**Development** **Context**	Eclipse Foundation	Eclipse Foundation	Fraunhofer IOSB	NOVA School of Science and Technology
**Programming** **Languages**	C# (.NET Core, Blazor, .NET Framework)	Java (primary), Python, C++, Rust, .NET	Java	Node-RED (low-code)
**Metamodel** **Support**	V3: Full	V3: Full	V3: Full	V3: Partial
**Licensing**	Apache 2.0 License	MIT License	Apache 2.0 License	EUPL v1.2 License
**Focus Areas**	Lightweight server implementation, companion app for AASX Package Explorer	Rich ecosystem, integration with many tools and visualization	AAS edge synchronization	No/low-code tools for GUI/dashboard
**Interface**	CLI, GUI	CLI, GUI	CLI	GUI
**Documentation Quality**	Moderate	Extensive	High	Low
**Dashboard**	✕	✓	✕	✓
**Docker Image**	✓	✓	✓	✓
**Data Monitoring**	✕	✓	✕	✓
**Import/Export AASX File**	Export only	✓	✕	✕
**Mobile Support**	✕	✓	✕	✕
**UI Semantic Identifier**	✕	✓	✕	✕
**AAS Editing Interface**	✕	✓	✕	✕

**Table 3:** Deployment and Utility Comparison of Open-Source AAS Solutions.

Deployment Aspect	AASX Server	Eclipse BaSyx	FA^3^ST Service	NOVAAS
Distribution Modes	• CLI executable (.NET console) • Docker image	• Java JAR (CLI) • Docker containers for each component • SDK	• Java JAR (CLI) • Docker image	• Docker image • Run by importing Node-RED flows
Startup Time	• Fast (<2 s) • Instant REST/OPC UA availability	• Moderate (a few seconds due to container startup and optional DB loading)	• Fast (<2 s) • Lightweight initialization	• Fast (<2 s) • Near-instant startup for small flows
CPU and Concurrency	• Multi-threaded • Low resource consumption	• Multi-threaded • Parallel integration (e.g., Camel routes)	• Multi-threaded • Efficient update propagation via internal bus	• Single-threaded event loop • May become CPU-bound under heavy simultaneous load
Scalability	• Vertical: Supports multiple AAS within per instance • Horizontal: Multiple instances possible; external discovery needed	• Vertical: Supports multiple AAS and multiple submodels per instance (multiple distinct shells) • Horizontal: Built-in registry supports multi-instance	• Vertical: Supports multiple submodels per instance • Horizontal: External registry required for multiple AAS instances	• Vertical: Supports multiple submodels per instance • Horizontal: External coordination required; best for small deployments
Edge/Device Deployment	• Suitable for industrial devices	• Java-based deployment on PCs and gateways	• Suitable for IoT gateways and high-end controllers	• Runs on Raspberry Pi and similar Linux-based gateways
Cloud Deployment	• Cloud-ready via Docker • Lacks multi-instance coordination without extra tools	• Cloud-friendly: modular services, Kubernetes deployment • Can integrate with cloud digital twin scenarios	• Cloud-ready via Docker • Can work in serverless environments; integrates with Eclipse Data Space connectors	• Primarily designed for on-prem/edge; not ideal for large-scale cloud production
Persistence	• In-memory by default; basic DB (SQLite) in v3	• Supports MongoDB or SQL for persistence • Offloads data from memory	• In-memory by default	• AAS state in-memory with importing on start
Remote Access	• Web UI accessible via browser	• Web UI can be hosted and accessed remotely for monitoring	• Remote access is via API	• Web dashboard accessible via browser
Access Control	• Supports OAuth2/OIDC token-based auth	• Integrates with OAuth2/JWT authentication	• No authentication by default (open API)	• Basic authentication for the web UI (username/password)

**Table 4:** Resource Utilization and Deployment Efficiency of Four AAS Implementations.

Open Source	Build Time	Image Size	Start-Up Time	Memory Usage (Idle)	CPU Usage (Idle)	Test Scenario (Load)	Average Memory Usage (Load)	Average CPU Usage (Load)
AASX Server	**25 s**	**627 MB**	~1 s	**88 MB**	0.6%	400 requests in 20 s	**~91.83 MB**	**~12.31%**
FA^3^ST Service	39 s	669 MB	~1 s	976 MB	**0.24%**	400 requests in 20 s	~798.08 MB	~84.59%
Eclipse BaSyx	127 s	~2.3 GB	57 s	1.28 GB	1.03%	400 requests in 20 s	~1.61 GB	~23.09%
NOVAAS	309 s	3.24 GB	~1 s	193.8 MB	1.1%	400 requests in 20 s	~241.15 MB	~16.89%

The tests were performed on hardware with Intel Core i7-1260P processor and 8 GB 3200 MHz RAM. 100% CPU usage is utilization of one core; if the CPU usage were 1600%, it would indicate that the container is fully utilizing all 16 cores. The “~” sign indicates approximate values.

## Data Availability

The materials used in the study are provided in Github repository [22].
